# Redox-Driven Blood–Nerve Barrier Dysfunction in Diabetic Peripheral Neuropathy: Mechanisms and Therapeutic Opportunities

**DOI:** 10.3390/antiox15060670

**Published:** 2026-05-26

**Authors:** Wei-Hsiu Huang, Chih-Shung Wong

**Affiliations:** 1 Department of Anesthesiology, Cathay General Hospital, Taipei 106, Taiwan; cgh17178@cgh.org.tw; 2 Graduate Institute of Medical Sciences, National Defense Medical University, Taipei 114, Taiwan

**Keywords:** diabetic peripheral neuropathy, blood–nerve barrier, peripheral nerve neurovascular unit, redox imbalance, oxidative stress, Nrf2, neuroinflammation, incretin-based therapy

## Abstract

Diabetic peripheral neuropathy (DPN) remains a leading cause of disability in diabetes, yet current care is largely symptomatic and does not directly address early neurovascular-immune pathology. This narrative review synthesizes clinical, redox, vascular, and immunological evidence into a peripheral nerve neurovascular unit (PNVU)/blood–nerve barrier (BNB)-centered framework for DPN. First, the review outlines the diagnostic and translational endpoint landscape of DPN, emphasizing that commonly used clinical, neurophysiological, small-fiber, and imaging-based tools capture important disease domains but do not directly assess early BNB dysfunction. It then reviews the anatomical and functional basis of the PNVU and BNB, including endoneurial microvascular endothelial cells, pericytes, basement membrane components, immune cells, and tight-junction proteins. Next, it discusses how chronic hyperglycemia and dyslipidemia drive metabolic-to-vascular coupling, redox imbalance, antioxidant defense failure, advanced glycation end products (AGEs), receptor for AGEs (RAGE), and nuclear factor-κB (NF-κB) signaling, endothelial activation, leukocyte recruitment, macrophage polarization, and junctional disassembly, culminating in increased BNB permeability and exposure of peripheral nerves to pro-inflammatory and neurotoxic mediators. Finally, it evaluates incretin-based therapies—including glucagon-like peptide-1 receptor agonists (GLP-1RAs), dipeptidyl peptidase-4 inhibitors (DPP-4 inhibitors, DPP-4is), and emerging multi-agonists—as potential modulators of oxidative and inflammatory stress within this framework. Although semaglutide and related agents show mechanistic plausibility and preclinical promise, direct evidence for incretin-mediated BNB stabilization in human DPN remains limited. By reframing DPN as a redox-driven neurovascular-immune disorder, this review highlights barrier-focused biomarkers, translational endpoints, and hypothesis-generating therapeutic opportunities that require clinical validation.

## 1. Introduction

Diabetes mellitus is among the most pressing global health challenges of the 21st century. A 2023 systematic analysis published in *The Lancet* projected that the global prevalence of diabetes would increase from 6.1% to 9.8% by 2050, corresponding to more than 1.31 billion affected individuals [1]. Diabetic peripheral neuropathy (DPN) is one of the most common and disabling chronic complications of diabetes, with a lifetime prevalence approaching 50% [2,3,4]. Painful DPN is also frequent; a recent systematic review and meta-analysis estimated a pooled prevalence of 33.9% among individuals with diabetes, with substantial heterogeneity across populations and study settings [5]. Clinically, DPN is associated with major downstream morbidity—including sensory loss, gait impairment, foot ulceration, and amputation risk [3]. Current therapies remain largely symptomatic and do not provide robust disease-modifying effects that halt or reverse underlying neurovascular-immune pathology [6]. This mismatch between clinical burden and disease-modifying options underscores the need for mechanistic frameworks that can connect clinical phenotypes with early biological events and actionable therapeutic targets.

Although contemporary diagnostic approaches integrate symptoms, signs, neurophysiological testing, small-fiber assessments, clinical scales, and exploratory imaging modalities [7,8], these tools mainly define disease phenotype and severity rather than directly assessing early blood–nerve barrier (BNB) dysfunction or peripheral nerve neurovascular unit (PNVU) stress in routine human studies. This gap is important because several established pathogenic mechanisms in DPN—including chronic hyperglycemia-driven metabolic injury, advanced glycation end product (AGE) formation, polyol pathway activation, oxidative stress, impaired antioxidant defenses, dyslipidemia, and inflammatory signaling—may converge on vascular and barrier dysfunction within the peripheral nerve microenvironment [9,10,11,12,13]. Recent transcriptomic and vascular/barrier-focused studies further support a broader view in which peripheral nerve injury arises from system-level dysfunction involving endoneurial endothelial cells, pericytes, basement membrane components, Schwann cells, axons, and resident or recruited immune cells within the PNVU [14,15,16,17,18]. Within this framework, diabetes-associated redox imbalance and impaired antioxidant defenses may create a permissive inflammatory milieu within peripheral nerves [12,13,14]. This milieu may promote endothelial activation and adhesion molecule induction, including changes in cell adhesion molecules (CAMs) and selectins associated with diabetic microvascular complications [19], and may further converge on leukocyte recruitment, macrophage accumulation, cytokine signaling, tight-junction disruption, and increased BNB permeability [20,21,22].

In this context, incretin-based therapies—particularly long-acting glucagon-like peptide-1 receptor agonists (GLP-1RAs) such as semaglutide—have attracted attention for potential benefits beyond glycemic control [23,24]. In addition to the SUSTAIN clinical program showing improvements in glycemic indices and cardiovascular outcomes [25,26,27], semaglutide and other GLP-1RAs have demonstrated anti-inflammatory, neuroprotective, or barrier-relevant effects in central nervous system models of neurodegeneration, stroke, subarachnoid hemorrhage, amyloid-related injury, and metabolic syndrome-associated blood–brain barrier dysfunction [28,29,30,31,32,33,34,35]. Preclinical evidence also suggests that semaglutide may alleviate diabetic neuropathic pain behaviors, in part by attenuating maladaptive neuroinflammatory signaling [36]. However, direct evidence that incretin-based therapies modify BNB integrity or barrier function in human DPN remains limited. This gap distinguishes mechanistic plausibility from clinically validated disease modification.

The contribution of this review is therefore not to propose an entirely new pathogenic element, but to synthesize existing clinical, redox, vascular, and immunological evidence into an integrated PNVU/BNB-centered framework for DPN. The review first maps the diagnostic and translational endpoint landscape of DPN and explains why conventional tools remain essential but insufficient for directly assessing early PNVU/BNB dysfunction. It then reviews the anatomical and functional basis of the PNVU and BNB, followed by redox imbalance, metabolic-to-vascular coupling, endothelial activation, macrophage polarization, and junctional disassembly as interconnected contributors to barrier dysfunction. Finally, it evaluates incretin-based therapies—including GLP-1RAs, dipeptidyl peptidase-4 inhibitors (DPP-4 inhibitors, DPP-4is), and emerging multi-agonists—as potential modulators of oxidative and inflammatory stress, while emphasizing current translational gaps, including the lack of validated BNB-specific biomarkers and limited direct human evidence for incretin-mediated BNB stabilization.

## 2. Literature Search and Selection Strategy

This narrative review was designed to synthesize clinical, mechanistic, and translational evidence linking diabetic peripheral neuropathy (DPN), blood–nerve barrier (BNB) dysfunction, peripheral nerve neurovascular unit (PNVU) stress, redox imbalance, neuroinflammation, and incretin-based therapeutic opportunities. Because the purpose of this review was integrative rather than systematic, no formal meta-analysis or risk-of-bias assessment was performed. Nevertheless, a structured literature search and selection strategy was used to improve transparency and reproducibility.

Relevant literature was identified through searches of PubMed, Web of Science, Scopus, and Google Scholar. The search strategy combined terms related to disease context, barrier biology, redox mechanisms, immune activation, diagnostic endpoints, and incretin-based therapies. Representative keywords included “diabetic peripheral neuropathy,” “diabetic neuropathy,” “blood-nerve barrier,” “peripheral nerve neurovascular unit,” “endoneurial microvascular endothelial cells,” “oxidative stress,” “redox imbalance,” “Nrf2,” “HO-1,” “SOD,” “neuroinflammation,” “macrophage polarization,” “tight junction,” “claudin-1,” “claudin-5,” “VCAM-1,” “ICAM-1,” “corneal confocal microscopy,” “intraepidermal nerve fiber density,” “GLP-1 receptor agonist,” “semaglutide,” “DPP-4 inhibitor,” “dual incretin agonist,” “tirzepatide,” and “biomarkers.” Additional articles were identified by screening the reference lists of relevant reviews, consensus statements, and key mechanistic studies.

Priority was given to peer-reviewed articles published in English, including clinical studies, experimental animal studies, in vitro mechanistic studies, consensus statements, guidelines, and recent narrative or systematic reviews. Recent publications from the past five years were prioritized when discussing emerging concepts, diagnostic biomarkers, redox signaling, incretin-based therapies, and translational endpoints. Earlier foundational studies were retained when they established widely accepted diagnostic criteria, barrier biology, oxidative stress mechanisms, or macrophage/neuroinflammatory pathways relevant to DPN and the PNVU.

Studies were included if they addressed at least one of the following domains: DPN clinical definition or phenotyping; diagnostic or translational endpoints; BNB or peripheral nerve barrier biology; PNVU structure or function; oxidative stress and antioxidant defense mechanisms; endothelial activation and immune-cell recruitment; macrophage polarization or neuroinflammation; tight-junction or basement membrane integrity; or incretin-based therapies with potential relevance to redox, vascular, immune, or barrier-related mechanisms. Studies were excluded if they were unrelated to diabetes, peripheral neuropathy, peripheral nerve biology, or translational mechanisms relevant to DPN. CNS-only studies were not used as direct evidence for BNB involvement but were considered when they provided mechanistic insight into GLP-1RA-associated redox, inflammatory, or barrier-related effects that may generate hypotheses for peripheral nerve research. Non-diabetic neuropathy studies were included only when they directly informed BNB biology, permeability assessment, immune trafficking, or methodological interpretation.

Given the narrative scope of this review, the included evidence was interpreted according to biological relevance and level of support. Clinical evidence, preclinical findings, in vitro mechanistic data, and hypothesis-generating interpretations were distinguished where appropriate, particularly when discussing incretin-mediated BNB stabilization and therapeutic opportunities. Claims based primarily on animal or CNS models were therefore presented as mechanistically plausible rather than clinically established in human DPN.

## 3. Clinical and Conceptual Rationale for a PNVU/BNB-Centered View of DPN

### 3.1. Diagnostic and Translational Endpoint Landscape in DPN

DPN encompasses a heterogeneous clinical spectrum, and available diagnostic or translational endpoints capture different levels of neuropathy biology rather than a single unified disease process [7,8]. Contemporary guidance and consensus statements therefore emphasize a staged approach that integrates symptoms, signs, and objective assessments, including the Toronto consensus framework for distal symmetric polyneuropathy (DSPN) [37,38,39].

Conventional bedside screening, vibration assessment, nerve conduction studies, and electromyography are most informative for large-fiber involvement and established neuropathy. In contrast, QST, IENFD, CCM, and autonomic or sudomotor testing provide complementary information on small-fiber-related sensory, structural, or autonomic abnormalities [7,40,41,42,43,44,45]. Clinical scales also serve different purposes: TCNS/mTCNS support screening, severity grading, and longitudinal clinical follow-up, whereas DN4 helps identify neuropathic pain phenotypes; however, these instruments remain partly dependent on patient reporting and examiner interpretation [46,47,48]. In research settings, DTI, HRUS, and MRN may further characterize nerve morphology or, for MRI-based approaches, microstructural alterations [49,50,51].

Taken together, these modalities are essential for clinical diagnosis, staging, phenotyping, and trial design, but they largely describe downstream neuropathy phenotypes rather than directly measuring early BNB dysfunction or PNVU stress in routine human studies. This mismatch provides the rationale for linking conventional DPN endpoints with upstream metabolic, vascular, redox, and inflammatory mechanisms in a PNVU/BNB-centered framework. A simplified overview of these diagnostic and translational endpoint modalities is provided in Table 1.

### 3.2. PNVU/BNB-Centered Conceptual Framework

Building on this endpoint gap, a PNVU/BNB-centered framework positions the BNB not simply as a passive anatomical barrier, but as an active regulatory interface through which systemic metabolic stress, redox imbalance, and inflammatory signaling may be translated into local peripheral nerve injury.

The traditional view of the BNB as a static, passive wall has evolved into the more dynamic concept of the PNVU. Like the neurovascular unit in the CNS, the PNVU represents a “functional syncytium” in which endoneurial microvascular endothelial cells (EMECs), pericytes, the basement membrane, and resident immune cells—particularly Iba1-positive macrophages—operate as an integrated system to maintain endoneurial homeostasis [15,16,17]. Rather than acting as a simple diffusion barrier, the BNB within the PNVU actively regulates the entry of circulating metabolites, inflammatory cues, and oxidative mediators, thereby shaping an immune-quiescent microenvironment that supports axonal and Schwann cell function [52].

Communication within this unit is bidirectional and highly context dependent. Endothelial cells couple vascular supply to neural metabolic demands, while pericytes and resident macrophages serve as “sentinels” that sense microenvironmental perturbations and provide trophic support through factors such as glial cell line-derived neurotrophic factor (GDNF) and vascular endothelial growth factor (VEGF) [17,53]. Under physiological conditions, this coordinated crosstalk buffers the endoneurial compartment against systemic fluctuations in circulating solutes and mediators and limits exposure to potentially neurotoxic signals. Conversely, when metabolic stress and redox imbalance persist, disruption of this coordination may predispose the BNB to endothelial activation, immune-cell recruitment, tight-junction disruption, increased permeability, and downstream neuroinflammatory amplification.

**Figure 1:**
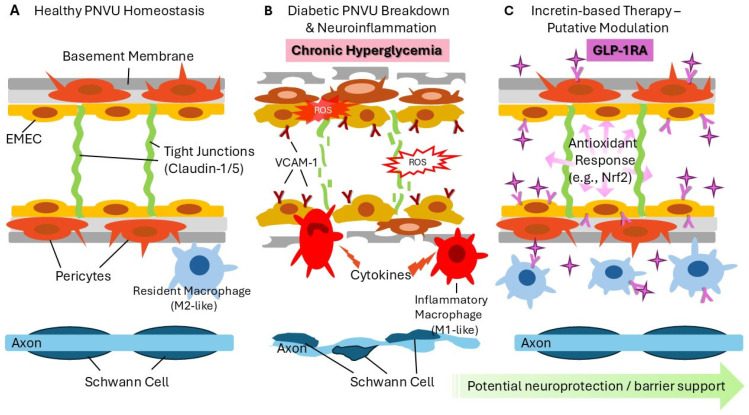
PNVU-centered conceptual framework for diabetic peripheral neuropathy and putative modulation by incretin-based therapies. (**A**) In health, endoneurial microvascular endothelial cells (EMECs), pericytes, and the basement membrane maintain tight-junction integrity and immune quiescence, supporting axonal and Schwann cell homeostasis. (**B**) In diabetes, chronic hyperglycemia promotes oxidative stress and endothelial activation (e.g., VCAM-1 upregulation), facilitating immune cell recruitment and pro-inflammatory cytokine release. These processes are associated with compromised barrier integrity, including tight-junction disruption, increased permeability, and PNVU destabilization. (**C**) Incretin-based therapies (illustrated by GLP-1 receptor agonists, GLP-1RAs) are proposed to attenuate oxidative and inflammatory signaling, induce antioxidant responses (e.g., Nrf2), and potentially support barrier stabilization and peripheral neuroprotection through improved metabolic milieu (glycemic control and weight/lipid effects) and possible GLP-1R-dependent vascular-immune actions within the PNVU/BNB. This schematic summarizes putative pathways and does not imply definitive causality or established clinical efficacy across all models. Abbreviations: BNB, blood–nerve barrier; DPN, diabetic peripheral neuropathy; EMEC, endoneurial microvascular endothelial cell; GLP-1RA, glucagon-like peptide-1 receptor agonist; Nrf2, nuclear factor erythroid 2-related factor 2; PNVU, peripheral nerve neurovascular unit; VCAM-1, vascular cell adhesion molecule 1.

A conceptual summary of this PNVU/BNB-centered framework is shown in Figure 1. In health, EMECs, pericytes, the basement membrane, resident macrophages, Schwann cells, and axons cooperate to maintain barrier integrity and endoneurial homeostasis. In diabetes, chronic hyperglycemia and signaling through the receptor for AGEs (RAGE), together with oxidative stress, may shift the PNVU toward endothelial activation and adhesion-molecule induction [9,10,11,19,22]. These changes may be accompanied by macrophage accumulation and inflammatory cytokine signaling and may contribute to tight-junction disruption, increased permeability, and barrier destabilization [14,20,21,22]. Incretin-based therapies, illustrated by GLP-1 receptor agonists, are considered in this review as putative modulators of oxidative and inflammatory stress within this framework, although direct evidence for clinically validated BNB stabilization in human DPN remains limited [23,24,36].

This conceptual framework provides the basis for the following sections. The anatomical and molecular architecture of the BNB is first reviewed, followed by comparative barrier biology, redox imbalance, metabolic-to-vascular coupling, endothelial activation, macrophage polarization, and junctional disassembly as interconnected contributors to BNB dysfunction in DPN.

### 3.3. Anatomical and Molecular Architecture of the BNB

The BNB is primarily composed of non-fenestrated EMECs, which are characterized by the presence of complex tight junction (TJ) proteins that seal the paracellular pathways [18,20]. These junctions are molecularly diverse, consisting of transmembrane proteins such as claudin-1, claudin-5, and occludin, which are anchored to the actin cytoskeleton via cytoplasmic scaffolding proteins like zonula occludens (ZO-1) [21,22].

Among these, claudin-5 is considered the “molecular gatekeeper” restricting paracellular diffusion of small solutes, while claudin-1 has been increasingly recognized for its role in maintaining high electrical resistance and barrier stability in the peripheral nervous system (PNS) [15]. Recent evidence highlights that the functional integrity of these molecular seals is not only dependent on total protein levels but also on their precise subcellular localization along the plasma membrane; chronic hyperglycemia often triggers the internalization and redistribution of these proteins, resulting in a characteristic “fragmented” staining pattern that is widely interpreted as barrier failure [54]. In the context of DPN, the delocalization or degradation of these specific proteins serves as a hallmark of barrier disintegration, and may precede overt clinical manifestations [16,55].

### 3.4. Comparative Barrier Biology: BNB Versus BBB

While both the BNB and the BBB share the common goal of neural protection, their cellular compositions differ significantly, which may explain the unique vulnerability of peripheral nerves in metabolic diseases. Unlike the BBB, which is enveloped by the foot processes of astrocytes (contributing to the glia limitans), the BNB lacks a comprehensive glial covering [17,19]. Instead, the BNB relies more heavily on a high density of pericytes and a layer of perivascular macrophages for structural and immunological reinforcement [56].

Furthermore, the permeability of the BNB is generally higher than that of the BBB, especially in areas such as the dorsal root ganglia (DRG), where the barrier is notably “leakier” and more permissive to circulating factors [19]. This lack of astrocytic support and higher baseline permeability renders the BNB particularly susceptible to chronic hyperglycemic insults and systemic inflammatory mediators circulating in the blood [57].

## 4. Redox Imbalance in the PNVU: Oxidative Damage, Antioxidant Defense Failure, and Metabolic-to-Vascular Coupling

### 4.1. The Landscape of Oxidative Damage in the PNVU

Chronic hyperglycemia does not merely increase the production of reactive oxygen species (ROS); it drives a broader state of redox imbalance within the PNVU through multiple converging mechanisms. In the diabetic milieu, excessive mitochondrial ROS generation, activation of NADPH oxidase-dependent pathways, and increased flux through the polyol, protein kinase C (PKC), and hexosamine pathways collectively intensify oxidative burden, while AGE-RAGE signaling further amplifies redox-sensitive inflammatory cascades [58,59,60]. Under physiological conditions, these insults are buffered by endogenous antioxidant defenses—including Nrf2-dependent transcriptional responses, superoxide dismutase (SOD), catalase, glutathione peroxidase (GPx), heme oxygenase-1 (HO-1), and the glutathione system—but in diabetes this protective network becomes progressively insufficient, allowing oxidative injury to accumulate across the molecular components of the PNVU [60,61]. A primary consequence of this metabolic insult is lipid peroxidation, marked by elevated levels of malondialdehyde (MDA) and 4-hydroxynonenal (4-HNE) [12,62]. These lipid-derived aldehydes cross-link with membrane proteins and disrupt the lipid bilayer of endoneurial endothelial cells. Beyond structural thinning, recent biophysical evidence indicates that oxidized bilayers are prone to the formation of transient hydrophilic pores, which directly contributes to the initial breach of the BNB and facilitates the paracellular leakage of neurotoxic macromolecules [62] (Figure 1B).

Furthermore, the diabetic environment promotes the formation of peroxynitrite, leading to the accumulation of nitrotyrosine—a hallmark of protein nitration that impairs vascular contractility and endothelial signaling [63,64]. This peroxynitrite-mediated damage is further exacerbated by the activation of signaling pathways such as Rho kinase (ROCK), which compromises the stability of the endothelial cytoskeleton and contributes to the disassembly of junctional complexes [63,65]. Simultaneously, DNA integrity within Schwann cells and axons is threatened by persistent oxidative stress, as evidenced by the accumulation of 8-hydroxy-2′-deoxyguanosine (8-OHdG) [66]. This oxidative DNA damage triggers mitochondrial-dependent apoptotic pathways, leading to a loss of Schwann cell support and exacerbating the pathological progression of DPN [66,67].

### 4.2. Metabolic Exhaustion of Endogenous Antioxidant Defenses: SOD, HO-1, and Impaired Nrf2 Signaling

To counter this oxidative deluge, the PNVU relies on an intricate hierarchy of defense mechanisms. Core components of this antioxidant network include SOD, which serves as the primary scavenger of superoxide radicals, and HO-1, a stress-inducible enzyme regulated by the Nrf2-ARE pathway that provides potent cytoprotective and anti-inflammatory effects [12,68]. Acting upstream of these enzymatic defenses, Nrf2 functions as a master redox-sensitive transcription factor that coordinates antioxidant and cytoprotective gene expression, thereby linking metabolic stress sensing to the preservation of endothelial and Schwann cell homeostasis [61,69].

In the persistent state of diabetes, however, these defense systems undergo a process of “metabolic exhaustion”. While there may be an initial compensatory upregulation in the early stages of hyperglycemia, persistent metabolic stress eventually blunts Nrf2 nuclear translocation and downstream transcriptional responses, contributing to the downregulation of SOD-2 and HO-1 protein expression [68,70]. As this compensatory network falters, the replenishment of glutathione-related and other antioxidant reserves becomes increasingly inadequate, shifting the PNVU toward a sustained pro-oxidant state [61,69,71]. This failure of the “endogenous shield” creates a state of redox imbalance, leaving the BNB defenseless against ROS and facilitating the transition from metabolic stress to structural disintegration and neuroinflammation [12,70].

### 4.3. Metabolic-to-Vascular Coupling: AGE-RAGE Signaling and Dyslipidemia as Upstream Drivers of PNVU Stress

**Figure 2:**
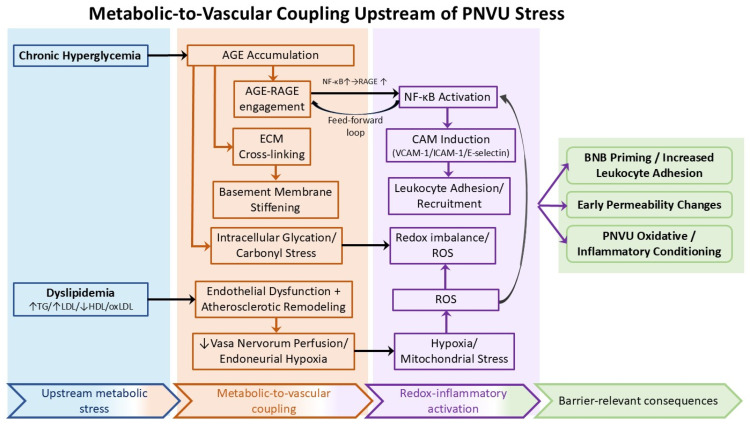
Metabolic-to-vascular coupling upstream of PNVU stress. Chronic hyperglycemia and dyslipidemia act as upstream metabolic drivers that converge on AGE-related signaling, redox imbalance, endothelial activation, and leukocyte recruitment. AGE accumulation contributes to extracellular matrix cross-linking, basement membrane stiffening, intracellular glycation/carbonyl stress, and AGE-RAGE engagement, whereas dyslipidemia promotes endothelial dysfunction, impaired vasa nervorum perfusion, and endoneurial hypoxia. These processes converge on ROS generation, NF-κB activation, adhesion molecule induction, and leukocyte adhesion/recruitment, thereby priming the BNB for early permeability changes and PNVU oxidative/inflammatory conditioning. The bottom arrow summarizes the proposed temporal progression from upstream metabolic stress to barrier-relevant consequences and is intended as a conceptual framework rather than a definitive sequence validated across all stages of human DPN. Abbreviations: PNVU, peripheral nerve neurovascular unit; AGE, advanced glycation end product; RAGE, receptor for advanced glycation end product; NF-κB, nuclear factor kappa B; VCAM-1, vascular cell adhesion molecule 1; ICAM-1, intercellular adhesion molecule 1; ROS, reactive oxygen species; BNB, blood–nerve barrier; TG, triglycerides; LDL, low-density lipoprotein; HDL, high-density lipoprotein; oxLDL, oxidized low-density lipoprotein.

Oxidative stress within the PNVU rarely arises in isolation; rather, it is primed by upstream metabolic cues that couple chronic hyperglycemia to vascular activation. These upstream metabolic-to-vascular coupling pathways are summarized in Figure 2.

A central driver is the cumulative burden of advanced glycation end products (AGEs), which can injure the microvasculature through (i) extracellular matrix cross-linking and basement membrane stiffening, (ii) intracellular glycation/carbonyl stress that perturbs cellular redox homeostasis, and (iii) receptor-mediated signaling through the receptor for AGEs (RAGE), which amplifies oxidative and inflammatory responses [72,73]. These AGE-dependent effects are not isolated lesions; rather, they converge on redox-sensitive endothelial activation and early barrier priming within the PNVU.

RAGE engagement triggers sustained nuclear factor-κB (NF-κB) activation and establishes a feed-forward loop in which RAGE expression and downstream inflammatory genes remain upregulated even after glycemic fluctuations. In endothelial cells, this program includes the induction of adhesion molecules (e.g., vascular cell adhesion molecule 1 (VCAM-1), intercellular adhesion molecule 1 (ICAM-1), E-selectin), increased leukocyte adhesivity, and a permissive proinflammatory phenotype—features that can “prime” the BNB for immune cell recruitment and permeability changes that later manifest as overt barrier breakdown [73,74,75]. By establishing a self-reinforcing circuit between NF-κB activation, further RAGE upregulation, and CAM induction, this axis helps maintain PNVU oxidative/inflammatory conditioning even when glycemic exposure fluctuates (Figure 2).

More importantly, dyslipidemia adds a vascular substrate that is frequently underemphasized in DPN mechanistic narratives. Clinical syntheses and meta-analytic data support associations between adverse lipid profiles and DPN, consistent with the idea that lipid-driven endothelial dysfunction and atherosclerotic remodeling can impair perfusion to the vasa nervorum and exacerbate endoneurial hypoxia [68,76,77]. Beyond epidemiologic association, dyslipidemia itself is increasingly recognized as a driver of diabetic microvascular dysfunction, and oxidized lipid stress may further intensify endothelial redox injury within this vascular niche [78,79]. Reduced nerve perfusion potentiates mitochondrial stress and lowers the threshold for endothelial activation, providing a mechanistic bridge from metabolic derangements to ROS amplification, NF-κB-CAM signaling, and neuroinflammatory priming within the PNVU (Figure 2).

## 5. Endothelial Activation and the Breach of Immune Privilege

### 5.1. Endothelial Dysfunction as a Gateway to BNB Breakdown

Building on the redox framework outlined in Section 4, oxidative stress and antioxidant defense failure translate chronic metabolic injury into an endothelial inflammatory phenotype, thereby converting the BNB from a homeostatic interface into a permissive site of immune activation. The physiological stability of the BNB is fundamentally dependent on the homeostatic state of EMECs. In the diabetic environment, chronic metabolic stress triggers endothelial dysfunction, characterized by a loss of vascular tone regulation and an increase in paracellular permeability [9,80]. This dysfunction transforms the BNB from a restrictive “gatekeeper” into a pro-inflammatory interface, facilitating the entry of systemic toxins and immune cells into the endoneurial space [80].

### 5.2. The NF-κB Pathway: Translating Metabolic Stress into Inflammation

A central mediator of this transformation is the nuclear factor-κB (NF-κB) signaling pathway. In this context, NF-κB functions as a redox-sensitive transcriptional hub through which persistent hyperglycemia, oxidative stress, and related danger signals are translated into endothelial inflammatory activation. Within the peripheral neurovascular unit, persistent hyperglycemia may activate inflammatory signaling pathways, including TLR4/NF-κB-related cascades in relevant endothelial and Schwann cell compartments, thereby promoting pro-inflammatory gene expression [81]. In diabetic neuropathy models, NF-κB activation has been implicated in inflammatory nerve injury, and its inhibition has been associated with improved nerve conduction and reduced thermal hyperalgesia [80].

### 5.3. The Adhesion Molecule Cascade: VCAM-1, ICAM-1, and Selectins

The hallmark of endothelial activation in DPN is the sequential upregulation of CAMs and selectins. Clinical follow-up studies have confirmed a significant association between elevated circulating levels of VCAM-1, ICAM-1, and E-selectin and the presence of DPN in type 2 diabetic patients [19]. Mechanistically, leukocyte recruitment proceeds in an ordered cascade—initial tethering/rolling followed by firm adhesion—driven by selectins and integrin-CAM interactions. Specifically, E-selectin is expressed on the vascular endothelium of peripheral nerves during acute metabolic or inflammatory stress, mediating the initial rolling of leukocytes along the BNB [82]. Subsequent upregulation of VCAM-1 and ICAM-1 then ensures firm leukocyte attachment to the endoneurial vessels. Histological evidence further indicates that increased expression of these molecules in the diabetic peripheral nerve precedes overt axonal degeneration, supporting their role as early pathological markers of barrier distress [19,83].

### 5.4. MCP-1/CCL2 and the Recruitment of Endoneurial Macrophages

The recruitment of immune cells across the compromised BNB is largely driven by Monocyte Chemoattractant Protein-1 (MCP-1/CCL2). As endothelial activation becomes sustained, chemokine signaling adds a second layer to CAM-dependent leukocyte trafficking by promoting directed monocyte recruitment across the increasingly permissive barrier. Recent research specific to the peripheral nervous system has identified that MCP-1 is significantly upregulated in the DRG and sciatic nerves following chronic metabolic insult [84]. This local production of MCP-1 creates a potent chemotactic gradient that guides CCR2-positive monocytes from the circulation into the endoneurial space, signifying the definitive loss of the BNB’s immune privilege [80,84].

## 6. Chronic Neuroinflammation and Macrophage Polarization

### 6.1. Resident vs. Recruited Macrophages: The Sentinel Shift

Within the redox-conditioned microenvironment established in the diabetic PNVU, macrophage accumulation is not merely a consequence of barrier dysfunction but an active amplifier of oxidative and inflammatory injury. Under physiological conditions, the PNVU maintains a lean population of resident Iba1-positive macrophages that serve as immunological “sentinels” located primarily in the perivascular space [56]. These cells are essential for sensing microenvironmental changes and providing trophic support to the BNB [56]. However, in the diabetic state, the upregulation of VCAM-1 and the secretion of MCP-1/CCL2 (discussed in Section 5) facilitate a massive influx of blood-derived monocytes [85,86]. This transition from a stable, resident population to a high-density, recruited population of Iba1-positive cells is a hallmark of DPN and serves as a primary driver of chronic neuroinflammation [86,87].

### 6.2. Macrophage Polarization: The M1/M2 Paradigm in the Diabetic Nerve

The pathological impact of Iba1-positive cells is determined by their polarization state. Importantly, redox imbalance not only promotes macrophage recruitment but also shapes macrophage polarization toward pro-inflammatory programs. In the persistent hyperglycemic environment, macrophages predominantly adopt the M1 (pro-inflammatory) phenotype. M1-like macrophage programs are associated with ROS production and secretion of pro-inflammatory cytokines, including tumor necrosis factor alpha (TNF-α), IL-1β, and IL-6 [88,89].

**Figure 3:**
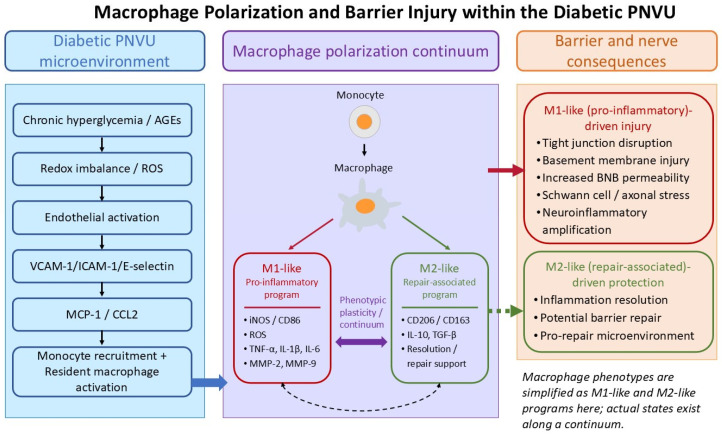
Macrophage Polarization and Barrier Injury within the Diabetic PNVU. In the diabetic peripheral nerve neurovascular unit (PNVU), chronic hyperglycemia, AGEs, redox imbalance, endothelial activation, adhesion molecule induction, and MCP-1/CCL2 signaling contribute to monocyte recruitment and resident macrophage activation. Within this redox-inflammatory microenvironment, macrophages may adopt predominantly M1-like pro-inflammatory or M2-like repair-associated programs, although actual phenotypes exist along a continuum. M1-like macrophages are associated with ROS production, pro-inflammatory cytokine release, and matrix-degrading activity, thereby contributing to tight-junction disruption, basement membrane injury, increased blood–nerve barrier (BNB) permeability, Schwann cell/axonal stress, and neuroinflammatory amplification. In contrast, M2-like macrophages are associated with inflammation resolution, potential barrier repair, and a pro-repair microenvironment. The schematic is intended as a conceptual framework and does not imply a rigid binary classification of macrophage states. Abbreviations: AGE, advanced glycation end product; BNB, blood–nerve barrier; CCL2, C-C motif chemokine ligand 2; DPN, diabetic peripheral neuropathy; ICAM-1, intercellular adhesion molecule 1; IL, interleukin; MCP-1, monocyte chemoattractant protein-1; MMP, matrix metalloproteinase; PNVU, peripheral nerve neurovascular unit; ROS, reactive oxygen species; TGF-β, transforming growth factor beta; TNF-α, tumor necrosis factor alpha; VCAM-1, vascular cell adhesion molecule 1.

Conversely, the M2 (anti-inflammatory) phenotype is associated with tissue repair and the secretion of neurotrophic factors. In DPN, a significant “polarization imbalance” occurs, where the M1 population significantly outnumbers the M2 population [88,90]. This skewed inflammatory milieu may sustain oxidative injury, impair barrier recovery, and promote ongoing axonal damage [89,90]. Notably, macrophage activation is plastic and exists along a continuum; thus, promoting a functional shift from M1-like to M2-like programs is hypothesized to support inflammation resolution and barrier/nerve repair (Figure 3).

### 6.3. The Cytokine Storm and Its Impact on Barrier Integrity

The accumulation of M1 macrophages leads to a localized “cytokine storm” within the endoneurium. In this setting, macrophage-derived cytokines and redox-active mediators act in concert to propagate barrier injury. Elevated levels of TNF-α and IL-1β have been shown to directly downregulate the expression of tight junction proteins in the endoneurial endothelium [55]. These cytokines trigger the internalization and subsequent degradation of claudin-5, effectively “dissolving” the molecular seal of the BNB [55,91]. Furthermore, activated macrophages release Matrix Metalloproteinases (MMPs), specifically MMP-2 and MMP-9, which enzymatically digest the vascular basement membrane [92]. This dual assault—the biochemical degradation of tight junctions and the physical destruction of the basement membrane—accelerates barrier destabilization and sets the stage for overt structural collapse [15,92].

## 7. Structural Collapse and Junctional Disassembly

### 7.1. The Molecular Gatekeepers: Claudin-1 and Claudin-5

Junctional disassembly represents the structural endpoint of persistent redox-inflammatory injury, in which oxidative stress, cytokine exposure, protease activation, and loss of perivascular support converge on the tight junction architecture of the BNB. The restrictive properties of the BNB are primarily governed by the TJ complex, a sophisticated protein network that seals the paracellular space between endoneurial endothelial cells. Among these, claudin-5 is recognized as the most critical isoform for regulating the permeability of small molecules and ions within the endoneurial microvasculature [15,93]. Furthermore, claudin-1, which is also expressed in the perineurium, contributes significantly to the overall paracellular resistance of the endoneurial barrier [93].

Recent research has emphasized that the maintenance of these gatekeepers is a multi-cellular effort within the PNVU. Specifically, pericyte-derived factors, such as GDNF, are essential for upregulating claudin-5 expression, highlighting that the structural integrity of the BNB is inextricably linked to the health of its supporting perivascular cells [55].

### 7.2. Junctional Disassembly and Protein Downregulation

In the diabetic PNVU, the “persistent redox-inflammatory assault” of chronic oxidative stress, pro-inflammatory cytokines, and MMPs culminates in the progressive disassembly of these tight junctions. Chronic hyperglycemia and the resulting inflammatory signaling—key components established in foundational DPN models—trigger the internalization and ubiquitin-mediated degradation of claudin-5 and claudin-1 [15,55].

Unlike acute injuries, the diabetic state induces a chronic attenuation of barrier tightness, characterized by junctional protein internalization and a fragmented immunostaining pattern [54]. This loss of junctional continuity represents the definitive structural failure of the BNB, effectively “dissolving” the molecular seal that protects the endoneurial microenvironment [54,55].

### 7.3. Assessing BNB Integrity: A Methodological Toolbox (Structural, Functional, and Cellular Readouts)

Because BNB breakdown is a multi-layered process—and reflects the cumulative consequence of redox stress, inflammatory trafficking, and structural remodeling—no single assay should be treated as a universal “gold standard.” A more rigorous approach is to triangulate (i) functional permeability, (ii) structural junction/ultrastructure and route-specific mechanisms, and (iii) endothelial immune activation and trafficking readouts, ideally within the same model and time window [18,94].

Functional permeability: Evans blue albumin leakage remains a convenient macromolecular tracer readout, but it is best presented as one option among complementary tracers and quantification strategies [95,96]. Fluorescent dextrans of defined molecular weights (e.g., 4–70 kDa) can probe size-selective leakage when circulation times and regional sampling are standardized [97]. Endogenous leakage markers (e.g., extravascular IgG/albumin or fibrinogen immunoreactivity) provide corroboration when paired with tracer assays [94,98]. For interpretability and reproducibility, permeability studies should report tracer identity/molecular weight, dose, circulation time, perfusion strategy, and tissue normalization (wet weight/protein).

Structural and route-specific mechanisms: Immunostaining or immunoblotting of tight-junction and scaffolding proteins (e.g., claudin-5/claudin-1, occludin, ZO-1) should be interpreted together with endothelial junctional patterns (continuous vs. fragmented) and, where feasible, TEM to visualize junctional clefts, basement-membrane changes, and vesicular density [18,94]. Where possible, route-specific mechanisms (paracellular vs. transcytotic pathways) should be distinguished to strengthen mechanistic attribution. Given evidence that baseline BNB “leakiness” relative to the BBB may be driven in part by higher transcytosis, quantifying caveolae/vesicle-associated pathways (e.g., caveolin-1) and fenestration-associated components such as PLVAP can strengthen mechanistic attribution when relevant [18,99]. Structural studies should specify regions of interest (endoneurium vs. perineurium; proximal vs. distal) and use blinded quantification whenever possible.

Cellular activation and trafficking: Barrier dysfunction often co-evolves with endothelial inflammatory activation; therefore, profiling adhesion molecules beyond VCAM-1 (e.g., ICAM-1 and selectins) and incorporating dynamic readouts such as leukocyte rolling/adhesion or perivascular cuffing can link adhesion signaling to barrier failure [19,94]. In parallel, macrophage accumulation, assessed using markers such as Iba1, CD68, or F4/80, should be interpreted together with phenotype-associated markers rather than as a simple binary readout. Markers linked to CD86/iNOS-like and CD206/CD163/CD204-like programs may help contextualize macrophage activation, but these states should be viewed as dynamic and overlapping rather than strictly dichotomous [18,89].

Collectively, prioritizing multi-readout designs (functional + structural + cellular) strengthens mechanistic interpretation from redox imbalance to barrier collapse and mitigates endpoint bias in BNB-centric DPN mechanisms [94,95]. Complementing the barrier-focused assays described above, Table 2 summarizes representative oxidative-stress and antioxidant readouts discussed in Section 4 that may help link redox imbalance to endothelial injury, barrier dysfunction, and neural vulnerability in DPN/PNVU studies.

## 8. Incretin-Based Therapies in DPN and the PNVU: Class Rationale, Semaglutide, and Translational Opportunities

### 8.1. Beyond Semaglutide: The Incretin Landscape (GLP-1RAs vs. DPP-4is; Emerging Multi-Agonists)

Although this review later focuses on semaglutide, the term “incretin-based therapies” is best supported when the broader incretin pharmacology is briefly mapped. Incretin-based agents include (i) GLP-1RAs that provide pharmacologic receptor activation, (ii) dipeptidyl peptidase-4 inhibitors (DPP-4i) that prolong endogenous GLP-1 (and other substrates), and (iii) emerging dual/triple agonists that combine incretin and related metabolic axes [101,102].

From a PNVU/BNB perspective, these classes differ not only in glycemic control potency but also in how they may interface with endothelial activation, immune trafficking, and barrier stress (Figure 1C). GLP-1RAs have shown anti-oxidative and anti-inflammatory effects in experimental neuropathy models, with reported improvements across behavioral, electrophysiologic, and structural readouts in peripheral nerves [103].

DPP-4i offer a complementary angle: beyond glucose lowering, DPP-4 inhibition has been reviewed as a potential modifier of diabetic microvascular complications, with experimental data spanning neuropathy and other microangiopathic endpoints [104]. In rodent neuropathy models, DPP-4i (e.g., sitagliptin, vildagliptin) were associated with improvements in nerve conduction and neuronal/DRG signaling, supporting the possibility that augmented incretin tone intersects with neurotrophic and inflammatory pathways relevant to barrier integrity [105,106]. In streptozotocin-induced diabetic neuropathic pain, teneligliptin has been reported to reduce neuroinflammatory signaling in the spinal dorsal horn and to engage antioxidant defense programs (e.g., Nrf2/HO-1), providing disease context support for incretin-linked anti-inflammatory/antioxidant actions [107]. Moreover, DPP-4 inhibition has been shown to reduce vascular leakage and inflammatory injury in other microvascular barriers (e.g., retina), providing mechanistic plausibility for barrier-stabilizing effects that merit direct testing at the BNB [108].

Emerging multi-agonists expand the landscape further. Dual GIP/GLP-1 receptor agonism with tirzepatide has demonstrated superior glycemic and weight-reduction efficacy compared with semaglutide in type 2 diabetes, while mechanistic reviews highlight its broader effects on insulin secretion, appetite regulation, and body weight reduction [109,110].

Positioning semaglutide within this landscape helps keep the section review-like rather than endpoint-driven: it clarifies what is known (class-level anti-inflammatory and anti-oxidative biology, along with strong metabolic effects) and what remains a gap (direct human DPN outcomes and BNB-centric readouts across incretin classes). The evidence landscape and putative PNVU/BNB targets are summarized in Table 3.

**Figure 4:**
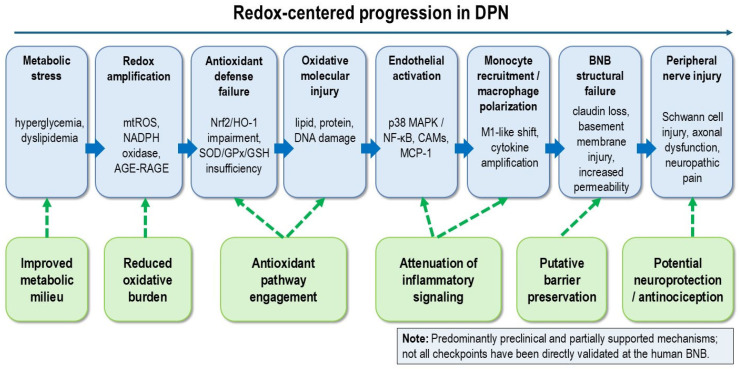
Redox-centered cascade linking metabolic stress to blood–nerve barrier (BNB) failure and putative incretin-responsive checkpoints. Chronic hyperglycemia and dyslipidemia promote mitochondrial reactive oxygen species (ROS) generation, NADPH oxidase activation, and AGE-RAGE signaling, which together amplify oxidative stress and overwhelm endogenous antioxidant defenses (e.g., Nrf2-HO-1, SOD, GPx, and glutathione-related systems). The resulting oxidative injury to lipids, proteins, and DNA contributes to endothelial activation, including p38 MAPK/NF-κB signaling, adhesion molecule induction, and chemokine upregulation, thereby facilitating monocyte recruitment, macrophage polarization toward pro-inflammatory programs, tight-junction disassembly, basement membrane injury, and increased barrier permeability. These events ultimately converge on Schwann cell and axonal injury within the peripheral nerve neurovascular unit (PNVU), contributing to neuropathic dysfunction in diabetic peripheral neuropathy (DPN). Incretin-based therapies, illustrated here by GLP-1 receptor agonists such as semaglutide, are hypothesized to intervene at multiple checkpoints by improving the metabolic milieu, attenuating oxidative and inflammatory signaling, engaging antioxidant pathways, and potentially preserving BNB structure and function. This schematic summarizes proposed and partially supported mechanisms and does not imply that all checkpoints have been directly validated at the human BNB.

Against this broader incretin landscape, the following subsections focus on semaglutide as a representative GLP-1RA with emerging preclinical evidence relevant to PNVU/BNB protection in DPN, while direct evidence for BNB restoration remains limited. The redox-centered pathogenic cascade and putative incretin-responsive checkpoints relevant to DPN and PNVU/BNB dysfunction are summarized in Figure 4.

### 8.2. GLP-1 Receptor Agonists, with Semaglutide as a Representative Agent: Pharmacological Properties and Antinociceptive Mechanisms

The therapeutic rationale for semaglutide in DPN is increasingly discussed as a multi-target process that extends beyond systemic glycemic control. GLP-1RAs may also exert direct antinociceptive effects by modulating GLP-1R-dependent signaling pathways within both the peripheral and central nervous systems. Recent pharmacological assessments emphasize the role of GLP-1R in pain disorders, highlighting its ability to regulate neuronal excitability and neuroinflammation [111]. In the context of DPN, these agents may address the complex clinical implications of neuropathic pain by targeting specific neurovascular pathways, thereby providing a plausible mechanistic framework for alleviating sensory symptoms while potentially influencing upstream disease-relevant processes [112].

### 8.3. Counteracting Metabolic Exhaustion: The Nrf2/Antioxidant Axis

Preclinical findings suggest that semaglutide may counteract aspects of the “metabolic exhaustion” associated with chronic hyperglycemic stress, particularly the progressive weakening of endogenous antioxidant defenses. Treatment has been shown to engage a cytoprotective response by activating the Nrf2 signaling pathway [12]. This activation is associated with the upregulation of downstream antioxidant enzymes, specifically HO-1 and SOD-2. By reinforcing antioxidant defenses and mitigating redox imbalance, semaglutide may help reduce ROS-related injury, thereby supporting endoneurial endothelial homeostasis and mitochondrial resilience under diabetic stress [112,113].

### 8.4. Attenuation of p38 MAPK/NF-κB Signaling and Neuroinflammation

A potential mechanism underlying the anti-inflammatory actions of GLP-1R agonism is the attenuation of p38 MAPK/NF-κB signaling. Chronic hyperglycemia and AGE-RAGE-related redox stress can activate inflammatory pathways and promote cytokine and adhesion-molecule expression [10]. In STZ-induced diabetic rats, GLP-1R agonist treatment attenuated sciatic-nerve inflammatory mediator expression, including TNF-α, IL-1β, MCP-1, and ICAM-1, together with reduced p38 MAPK/NF-κB activation [114]. These findings support the plausibility that GLP-1R agonism may reduce peripheral nerve inflammatory signaling and endothelial-immune cell interactions, although direct evidence for semaglutide-mediated protection of the human BNB remains limited. In addition, semaglutide has been reported to attenuate neuroinflammation in the spinal cord dorsal horn, suggesting a potential contribution to reduced central sensitization in diabetic neuropathic pain [36].

### 8.5. Putative Effects on BNB Structure and Function

A central translational question is whether attenuation of upstream oxidative and inflammatory stressors by semaglutide can translate into structural stabilization and functional preservation of the BNB. Rather than directly demonstrating BNB restoration, current evidence supports the plausibility that GLP-1RA therapy may help preserve tight-junction organization and barrier selectivity under diabetic stress. By mitigating biochemical insults through the aforementioned antioxidant and anti-inflammatory pathways, semaglutide may reduce the internalization and degradation of the tight junction complex. By attenuating upstream oxidative and inflammatory stressors, GLP-1RA therapy may create conditions that favor maintenance or partial recovery of tight-junction organization and barrier selectivity, although direct evidence at the human BNB remains limited. Preclinical findings suggest that semaglutide may help preserve or normalize the membrane localization and total protein levels of claudin-1 and claudin-5, thereby supporting the molecular seal that defines the endoneurial microenvironment [55]. Consistent with this possibility, functional permeability may be assessed by reduced macromolecular tracer leakage (e.g., Evans blue-albumin extravasation or fluorescent dextrans), which may indicate a shift from a pathological “leaky” barrier toward a more restrictive interface [95,97]. Clinical and preclinical observations further suggest that GLP-1RA therapy may be associated with reduced pathological nerve swelling and partial improvement of structural and functional abnormalities in chronic DPN [114,115].

## 9. Conclusions

The integrity of the BNB is a fundamental prerequisite for peripheral nerve homeostasis. As highlighted throughout this review, DPN progression is closely linked to PNVU dysfunction, where disruption of the neurovascular–immune axis drives the transition from metabolic stress to structural injury. The sequential cascade—redox imbalance and exhaustion of endogenous antioxidant defenses (including the Nrf2/HO-1 axis), followed by NF-κB-mediated endothelial activation (e.g., VCAM-1 upregulation), and subsequent disassembly of claudin-5 and claudin-1 tight junctions—provides a mechanistic roadmap for hypothesis-driven therapeutic investigation. Viewed through this redox-centered framework, DPN may be understood not only as a distal axonopathy but also as a neurovascular disorder shaped by oxidative stress, antioxidant defense failure, and inflammatory barrier injury within the PNVU.

Incretin-based therapies, particularly long-acting GLP-1 receptor agonists such as semaglutide, represent promising candidates for further investigation in DPN. By potentially attenuating oxidative stress and neuroinflammation, these agents may contribute to preservation or functional stabilization of the BNB in preclinical settings—with experimental readouts consistent with reduced barrier permeability (e.g., reduced Evans Blue extravasation) and preservation of the structural continuity of the endoneurial seal, including tight-junction proteins such as claudin-1 and claudin-5 [58,90,92]. Emerging clinical and preclinical observations further suggest improvements in nerve swelling and dysfunction with GLP-1RA therapy [114,115], supporting the hypothesis that incretin-based therapies may have disease-modifying potential beyond glucose lowering alone, which requires validation in adequately powered human studies.

## 10. Future Perspectives

### 10.1. Knowledge Gaps and Unresolved Questions

Several knowledge gaps currently limit the translation of a PNVU/BNB-centered framework into clinical DPN research. First, DPN is biologically and clinically heterogeneous. Disease stage, metabolic profile, diabetes duration, painful versus painless phenotypes, small- versus large-fiber predominance, and the degree of neurovascular involvement may all influence whether redox imbalance, endothelial activation, immune-cell recruitment, or barrier dysfunction is the dominant pathogenic driver in a given patient subgroup. Future studies should therefore avoid treating DPN as a single uniform endpoint and should instead incorporate phenotype- and stage-stratified designs.

Second, mechanistic attribution remains uncertain. It is unclear whether putative neurovascular benefits of incretin-based therapies are driven primarily by direct actions on the peripheral nervous system/PNVU or indirectly through improvements in glycemia, weight, lipids, and systemic inflammation. Preclinical data support the plausibility of direct GLP-1R signaling in peripheral nerves and Schwann cells [116,117], and small human neurophysiology studies have reported improved peripheral nerve excitability and axonal function over approximately 3 months, with changes not fully explained by concurrent HbA1c reductions [118,119]. However, available human datasets remain small, heterogeneous, and often underpowered for neuropathy or neurovascular endpoints. Notably, an 18-month proof-of-concept open-label randomized study reported no statistically significant between-group differences in multiple neuropathy measures with exenatide despite similar glycemic control [120]. These findings highlight the need to distinguish direct PNVU/BNB actions from indirect metabolic effects.

Third, clinically feasible BNB-specific biomarkers remain poorly standardized. Direct assessment of BNB permeability or endoneurial microvascular morphology is difficult in human studies, and commonly used endpoints such as NCS, QST, IENFD, CCM, and nerve imaging each capture only partial aspects of neuropathy biology. Circulating endothelial or inflammatory markers, including sVCAM-1, sICAM-1, E-selectin, cytokine profiles, and oxidative stress markers, may provide complementary information, but their specificity for BNB dysfunction remains uncertain. As emphasized in recent reviews and meta-analytic work on GLP-1RAs and DPN, future studies should incorporate more standardized neuropathy endpoints and biomarker strategies to clarify therapeutic effects beyond glycemic control [113,121].

Finally, direct clinical evidence for incretin-mediated BNB stabilization remains limited. Although preclinical and CNS-barrier studies support mechanistic plausibility, these findings cannot be assumed to translate directly to the human BNB. Long-term prospective studies are needed to determine whether GLP-1RAs, DPP-4 inhibitors, or emerging multi-agonists can modify neurovascular function, barrier-related biomarkers, or structural nerve outcomes beyond improvements in glycemia, weight, lipids, and systemic inflammation. Future translational efforts should also clarify how redox-focused biomarkers can be integrated with BNB/PNVU readouts across experimental and clinical settings.

### 10.2. Key Research Questions

Key priorities for the next phase of translational research include the following:

(i) Mechanism and primary target compartment
  - Is any neuroprotection independent of metabolic improvement?
  - Which PNVU compartment is the primary target (endothelium, Schwann cells, immune cells)?
(ii) Translational endpoints and biomarkers
  - Which outcomes provide the most reliable animal-to-human bridges (NCV, QST, IENFD, MR neurography, circulating endothelial/immune biomarkers)?
  - Which BNB/PNVU biomarker panels are feasible, repeatable, and responsive in clinical trials?
  - How should redox-focused readouts (e.g., lipid peroxidation, oxidative DNA damage, antioxidant pathway engagement) be incorporated alongside barrier-focused biomarkers?
(iii) Therapeutic class effects and next-generation incretins
  - Do DPP-4is and emerging multi-agonists (dual/triple agonists) provide additive or distinct benefits versus GLP-1RAs for neuropathy outcomes [122]?
(iv) Dose, duration, and phenotype enrichment
  - What duration and dosing are required for structural recovery (e.g., barrier integrity, axonal regeneration)?
  - Are there patient subgroups (phenotypes) most likely to benefit?

### 10.3. Translational Roadmap for Clinical Studies

Future trials and longitudinal cohorts should ideally use stage-stratified and phenotype-enriched designs, with predefined multimodal endpoints that capture symptoms, large- and small-fiber function, structural nerve integrity, neurovascular status, endothelial activation, inflammatory signaling, and oxidative stress biology over sufficiently long follow-up periods. To advance BNB/PNVU-targeted strategies toward human DPN, future studies should prioritize:

1. Clinical validation: Adequately powered clinical studies are needed to confirm translation to human DPN. Because direct assessment of BNB permeability and endoneurial morphology is invasive, validation will likely rely on scalable surrogate endpoints (e.g., IENFD/skin biopsy, corneal confocal microscopy, nerve imaging such as MRN/DTI/HRUS, and functional testing including NCS/EMG and QST; Table 1).
2. Timing of intervention: Determine whether GLP-1RAs are most effective when initiated in early-stage DPN (prevention) or whether they can reverse established chronic barrier dysfunction (repair). Stage-stratified designs and longitudinal follow-up using structural/functional endpoints (e.g., CCM/IENFD and NCS/QST) will help distinguish prevention from recovery.
3. Biomarker development: Develop non-invasive, high-resolution imaging (e.g., dynamic contrast-enhanced MRI/MR neurography as a proxy of microvascular permeability) and blood-based panels for real-time monitoring of BNB integrity. Candidate readouts include endothelial activation/barrier stress markers (e.g., sVCAM-1/sICAM-1 and exploratory tight-junction-related proteins such as claudin-5/occludin/ZO-1), downstream nerve injury markers (e.g., serum neurofilament light chain), and complementary structural surrogates (e.g., corneal confocal microscopy). MOA-oriented panels should also consider indirect metabolic effects (e.g., HbA1c/CGM metrics, circulating AGE/sRAGE) when interpreting biomarker changes. Where feasible, biomarker frameworks should also incorporate redox-focused readouts—such as markers of lipid peroxidation, protein nitration/oxidation, oxidative DNA damage, and endogenous antioxidant pathway engagement—to better connect barrier dysfunction with oxidative stress biology.
4. Multimodal approaches: Evaluate rational combinations that target non-overlapping nodes (including metabolic stress, oxidative injury, and neuroinflammation), such as GLP-1RAs paired with SGLT2 inhibitors or agents that modulate antioxidant defense pathways, including Nrf2-linked signaling, while monitoring safety and tolerability in DPN populations.

### 10.4. Closing Perspective

Ultimately, shifting the therapeutic focus toward BNB/PNVU preservation and repair-oriented strategies may provide a useful translational framework for developing interventions aimed at reducing the debilitating sensory and motor deficits associated with diabetic neuropathy. From a redox-centered perspective, future progress will depend on linking oxidative-stress biology, barrier dysfunction, and peripheral nerve injury within a coherent translational framework.

## Figures and Tables

**Table 1:** Clinical and translational endpoint modalities for linking DPN phenotypes to PNVU/BNB-focused research.

Modality/Representative References	Primary Domain/Fiber Type	Most Informative Use or Stage	Key Limitation
Bedside screening: 10 g monofilament; vibration/VPT [7,38]	Large-fiber function; loss of protective sensation	Rapid screening; established or advanced DPN; ulcer-risk stratification	Low sensitivity for early small-fiber neuropathy
Clinical scales: TCNS/mTCNS [46,47]; DN4 [48]	Symptoms, signs, severity, and pain phenotype	Screening, phenotyping, and longitudinal monitoring	Subjective components; inter-rater variability
NCS/EMG [39,45]	Large-fiber function	Objective confirmation; DSPN staging; trial eligibility	May miss isolated small-fiber disease
QST/sudomotor testing [7,42,43,44,45]	Small-fiber sensory/autonomic function	Early or painful DPN; functional phenotyping	Cooperation-, protocol-, site-, and device-dependent
IENFD skin biopsy [40,45]	Small-fiber structure	Early small-fiber loss; longitudinal structural assessment	Invasive; processing- and sampling-site dependent
Corneal confocal microscopy (CCM) [41]	Small-fiber structure; corneal nerve morphology	Noninvasive small-fiber structural assessment; longitudinal monitoring	Requires equipment and standardized image analysis
DTI [49]/HRUS [50]/MRN [51]	Nerve morphology; structural or microstructural changes	Exploratory phenotyping; research endpoint	Cost, access, and limited standardization

Abbreviations: CCM, corneal confocal microscopy; DN4, Douleur Neuropathique 4; DPN, diabetic peripheral neuropathy; DSPN, distal symmetric polyneuropathy; DTI, diffusion tensor imaging; EMG, electromyography; HRUS, high-resolution ultrasound; IENFD, intraepidermal nerve fiber density; MRN, magnetic resonance neurography; NCS, nerve conduction studies; QST, quantitative sensory testing; TCNS, Toronto Clinical Neuropathy Score; VPT, vibration perception threshold.

**Table 2:** Representative oxidative stress and antioxidant readouts relevant to mechanistic studies of DPN and PNVU dysfunction.

Domain/Representative References	Representative Readouts	Biological Significance	Typical Methods
Lipid peroxidation [58,62]	MDA; 4-HNE	Membrane lipid oxidation; aldehyde-mediated endothelial and neural injury	TBARS; HPLC; ELISA; IHC/IF; immunoblotting
Protein oxidation/nitration [63,64]	Nitrotyrosine; protein carbonyls	Protein nitration and oxidative modification; impaired vascular and cellular signaling	IHC/IF; immunoblotting; DNPH-based assays
Oxidative DNA damage [66,67,100]	8-OHdG	Nuclear or mitochondrial DNA oxidation; Schwann cell vulnerability and apoptosis-related injury	ELISA; IHC/IF; HPLC; LC-MS
Antioxidant response/defense failure [60,61,68,69,70,71]	Nrf2 nuclear translocation; HO-1; SOD1/2; catalase; GPx; GSH/GSSG ratio	Antioxidant pathway activation or exhaustion; redox-buffering capacity	Fractionation assays; immunoblotting; RT-qPCR; enzyme activity assays; glutathione assays
Mitochondrial stress/dysfunction [58,60,66,67,100]	mtROS; mitochondrial membrane potential; respiratory enzyme/OXPHOS changes; ATP content	ROS amplification; bioenergetic failure; Schwann cell and axonal vulnerability	MitoSOX; JC-1/TMRE/TMRM; respirometry; ATP assays; TEM

Abbreviations: 4-HNE, 4-hydroxynonenal; 8-OHdG, 8-hydroxy-2′-deoxyguanosine; DNPH, 2,4-dinitrophenylhydrazine; DPN, diabetic peripheral neuropathy; GPx, glutathione peroxidase; GSH/GSSG, reduced/oxidized glutathione ratio; HO-1, heme oxygenase-1; HPLC, high-performance liquid chromatography; IHC/IF, immunohistochemistry/immunofluorescence; LC-MS, liquid chromatography–mass spectrometry; MDA, malondialdehyde; mtROS, mitochondrial reactive oxygen species; Nrf2, nuclear factor erythroid 2-related factor 2; OXPHOS, oxidative phosphorylation; PNVU, peripheral nerve neurovascular unit; SOD, superoxide dismutase; TBARS, thiobarbituric acid reactive substances; TEM, transmission electron microscopy.

**Table 3:** Incretin-based therapies—evidence levels and putative actions on the PNVU and BNB.

Class	Representative Agents	Evidence in DPN	Putative PNVU/BNB-Relevant Actions	Key Limitations/Notes
GLP-1 receptor agonists (GLP-1RAs)	Liraglutide; semaglutide; exenatide	Preclinical: reported improvements in nerve function and reduced oxidative/inflammatory injury in multiple models [103].	Endothelial anti-oxidative and anti-inflammatory signaling; reduced neuroimmune activation; potential preservation of tight-junction integrity through reduced inflammatory stress.	Human evidence for DPN outcomes is still limited/heterogeneous; class effects vs. agent-specific effects require clarification.
DPP-4 inhibitors (DPP-4is)	Sitagliptin; vildagliptin; linagliptin	Preclinical: reported improvements in NCV/IENFD and DRG signaling in diabetic rodents [105,106]. Clinical: preliminary data for microvascular benefit [104].	Augments endogenous GLP-1 and affects non-incretin substrates; may reduce endothelial inflammation and vascular leakage in other barriers (e.g., retina) [108].	Direct BNB studies are sparse; effects may be partly mediated by improved glycemia; need DPN trials with barrier-relevant endpoints.
Dual incretin agonists (GIP/GLP-1)	Tirzepatide	Strong glycemic/weight efficacy vs semaglutide [109]; broader metabolic rationale [110]; neuropathy endpoints largely untested.	Metabolic unloading (glucose, lipids, weight) may reduce upstream PNVU stressors (AGE-RAGE, dyslipidemia/perfusion impairment) and secondarily attenuate endothelial activation.	Translation to DPN/BNB remains hypothesis-driven; microvascular inflammation/perfusion effects need dedicated studies.

Abbreviations: BNB, blood–nerve barrier; DPN, diabetic peripheral neuropathy; PNVU, peripheral nerve neurovascular unit; GLP-1RA(s), GLP-1 receptor agonist(s); DPP-4i(s), dipeptidyl peptidase-4 inhibitor(s); GIP, glucose-dependent insulinotropic polypeptide; DRG, dorsal root ganglion; NCV, nerve conduction velocity; IENFD, intraepidermal nerve fiber density.

## Data Availability

No new data were created or analyzed in this study.
